# Bis(4,4′-methyl­enedianilinium) naphthalene-1,5-disulfonate dihydrate

**DOI:** 10.1107/S160053680800723X

**Published:** 2008-03-20

**Authors:** Lin-Heng Wei

**Affiliations:** a College of Environment and Planning, Henan University, Kaifeng 475001, People’s Republic of China

## Abstract

The asymmetric unit of the title salt, C_13_H_16_N_2_^2+^·C_10_H_6_O_6_S_2_^2−^·2H_2_O, consists of one dication located on a general position, half each of two centrosymmetric dianions, and two uncoordinated water mol­ecules in general positions. In the dication, the dihedral angle between the benzene rings is 74.67 (6)°. The cations and anions inter­act through N—H⋯O hydrogen bonds. The NH_3_^+^ functional groups are also involved in N—H⋯O hydrogen bonds with the water mol­ecules, forming an infinite three-dimensional framework in the crystal structure.

## Related literature

For related literature, see: Wang & Wei (2007 ▶).
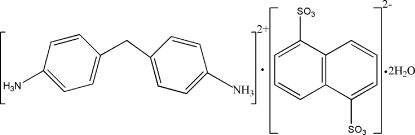


## Experimental

### 

#### Crystal data

C_13_H_16_N_2_^2+^·C_10_H_6_O_6_S_2_^2−^·2H_2_O
*M* *_r_* = 522.58
Triclinic, 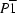

*a* = 7.9652 (6) Å
*b* = 10.9135 (8) Å
*c* = 13.8158 (10) Å
α = 87.429 (1)°
β = 85.820 (1)°
γ = 83.262 (1)°
*V* = 1188.72 (15) Å^3^
*Z* = 2
Mo *K*α radiation
μ = 0.28 mm^−1^
*T* = 296 (2) K
0.13 × 0.10 × 0.08 mm

#### Data collection

Bruker SMART APEX CCD area-detector diffractometer
Absorption correction: multi-scan (*SADABS*; Sheldrick, 2003 ▶) *T*_min_ = 0.966, *T*_max_ = 0.978
12513 measured reflections
4644 independent reflections
3999 reflections with *I* > 2σ(*I*)
*R*_int_ = 0.015

#### Refinement

*R*[*F* ^2^ > 2σ(*F* ^2^)] = 0.039
*wR*(*F* ^2^) = 0.117
*S* = 1.08
4644 reflections
334 parameters
30 restraints
H atoms treated by a mixture of independent and constrained refinement
Δρ_max_ = 0.30 e Å^−3^
Δρ_min_ = −0.30 e Å^−3^

### 

Data collection: *SMART* (Bruker, 2003 ▶); cell refinement: *SAINT-Plus* (Bruker, 2003 ▶); data reduction: *SAINT-Plus*; program(s) used to solve structure: *SHELXS97* (Sheldrick, 2008 ▶); program(s) used to refine structure: *SHELXL97* (Sheldrick, 2008 ▶); molecular graphics: *PLATON* (Spek, 2003 ▶); software used to prepare material for publication: *PLATON*.

## Supplementary Material

Crystal structure: contains datablocks global, I. DOI: 10.1107/S160053680800723X/bh2163sup1.cif

Structure factors: contains datablocks I. DOI: 10.1107/S160053680800723X/bh2163Isup2.hkl

Additional supplementary materials:  crystallographic information; 3D view; checkCIF report

## Figures and Tables

**Table 1:** Hydrogen-bond geometry (Å, °)

*D*—H⋯*A*	*D*—H	H⋯*A*	*D*⋯*A*	*D*—H⋯*A*
N1—H1*B*⋯O1^i^	0.89	1.91	2.770 (2)	162
N1*A*—H1*A*1⋯O5^ii^	0.89	2.03	2.897 (3)	163
N1—H1*C*⋯O1*W*^i^	0.89	2.07	2.948 (3)	167
N1*A*—H1*A*2⋯O2*W*^iii^	0.89	1.86	2.739 (3)	171
N1*A*—H1*A*3⋯O1*W*^iii^	0.89	1.95	2.807 (3)	161
O1*W*—H1*WA*⋯O4^i^	0.860 (10)	1.801 (10)	2.645 (2)	166 (2)
O1*W*—H1*WB*⋯O6^iv^	0.854 (10)	1.957 (11)	2.807 (2)	174 (3)

Symmetry codes: (i) 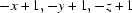; (ii) 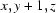; (iii) 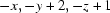; (iv) 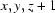.

**Fig. 1.**
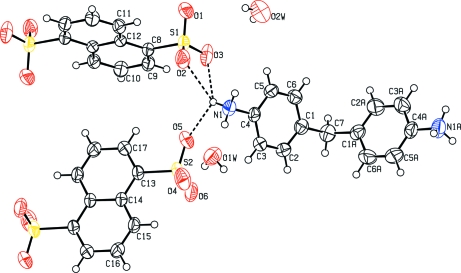
The structure of (I) including the asymmetric unit (labeled atoms) and anions completed through symmetry operators: unlabeled atoms in the C10 anion are related to labeled atoms by symmetry code -x + 1, -y, -z + 1; unlabeled atoms in the C16 anion are related to labeled atoms by symmetry code -x + 1, -y, -z. Displacement ellipsoids for non-H atoms are drawn at the 30% probability level. Hydrogen bonds are shown as dashed lines.

**Fig. 2.**
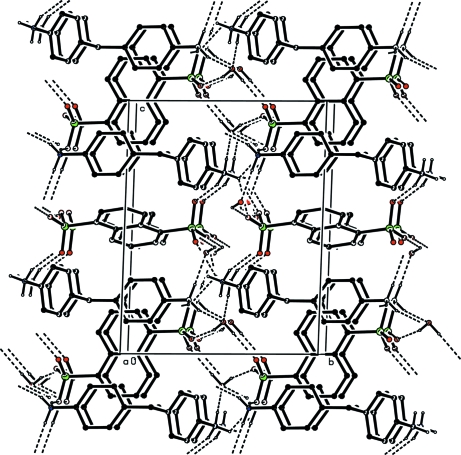
The crystal packing of (I). Hydrogen bonds are shown as dashed lines. For clarity, H atoms not involved in hydrogen bonds are omitted.

## supplementary crystallographic information

### Comment

This work continues our previous synthetic and structural studies of supramolecular interactions in aromatic molecular salts and adducts (Wang & Wei, 2007). Herein we report the structure of the title salt, (I).

The title complex, (I), consists of one crystallographically independent 4,4'-diphenylmethylendiammonium dication, two water molecules and two independent half naphthalene-1,5-disulfonate dianions. In the dication, the dihedral angle between benzene rings is 74.67 (6)°, and central C1—C7—C1A angle is 112.23 (16)° (Fig. 1). Each dianion is placed on an inversion centre. The 4,4'-diphenylmethylendiammonium dication interact with two naphthalene-1,5-disulfonate dianions through N—H···O hydrogen bonds. These units are further linked by water molecules into an infinite three-dimensional framework by hydrogen bonds (Fig. 2).

### Experimental

A 5 ml e thanol solution of 4,4'-methylene-bis(benzenamine) (0.5 mmol, 0.10 g) was added to an aqueous solution (25 ml) of naphthalene-1,5-disulfonic acid (0.50 mmol, 0.15 g). The mixture was stirred for 10 min. at 373 K. The solution was filtered, and the filtrate was allowed to stand at room temperature. After several days, colourless crystals suitable for X-ray diffraction were obtained.

### Refinement

H atoms for water molecules O1W and O2W were located in a difference map and refined with a geometry regularized through restrictions for distances: O—H = 0.85 (1) and H···H = 1.34 (1) Å. In order to reduce isotropic displacement parameters for water H atoms, *SIMU *restraints (similar *U*_ij_ components; Sheldrick, 2008) were applied for water molecules. Other H atoms were placed in calculated positions with bond lengths fixed to N—H = 0.89, C—H = 0.93 (aromatic CH) and C—H = 0.97 Å (methylene CH2 group) and were refined as riding atoms, with *U*_iso_(H) = 1.5 *U*_eq_(carrier N) or *U*_iso_(H) = 1.2 *U*_eq_(carrier C).

### Crystal data

**Table:**

C_13_H_16_N_2_^2+^·C_10_H_6_O_6_S_2_^2–^·2H_2_O	*Z* = 2
*M**_r_* = 522.58	*F*_000_ = 548
Triclinic, *P*1	*D*_x_ = 1.460 Mg m^−^^3^
Hall symbol: -P 1	Mo *K*α radiation λ = 0.71073 Å
*a* = 7.9652 (6) Å	Cell parameters from 3560 reflections
*b* = 10.9135 (8) Å	θ = 2.3–28.5º
*c* = 13.8158 (10) Å	µ = 0.28 mm^−^^1^
α = 87.429 (1)º	*T* = 296 (2) K
β = 85.820 (1)º	Block, colourless
γ = 83.262 (1)º	0.13 × 0.10 × 0.08 mm
*V* = 1188.72 (15) Å^3^	

### Data collection

**Table:**

Bruker SMART APEX CCD area-detector diffractometer	4644 independent reflections
Radiation source: fine-focus sealed tube	3999 reflections with *I* > 2σ(*I*)
Monochromator: graphite	*R*_int_ = 0.015
*T* = 296(2) K	θ_max_ = 26.0º
ω scans	θ_min_ = 1.9º
Absorption correction: multi-scan(SADABS; Sheldrick, 2003)	*h* = −9→9
*T*_min_ = 0.966, *T*_max_ = 0.978	*k* = −13→13
12513 measured reflections	*l* = −17→17

### Refinement

**Table:**

Refinement on *F*^2^	Secondary atom site location: difference Fourier map
Least-squares matrix: full	Hydrogen site location: inferred from neighbouring sites
*R*[*F*^2^ > 2σ(*F*^2^)] = 0.039	H atoms treated by a mixture of independent and constrained refinement
*wR*(*F*^2^) = 0.117	*w* = 1/[σ^2^(*F*_o_^2^) + (0.0622*P*)^2^ + 0.4195*P*] where *P* = (*F*_o_^2^ + 2*F*_c_^2^)/3
*S* = 1.08	(Δ/σ)_max_ < 0.001
4644 reflections	Δρ_max_ = 0.30 e Å^−^^3^
334 parameters	Δρ_min_ = −0.30 e Å^−^^3^
30 restraints	Extinction correction: none
Primary atom site location: structure-invariant direct methods	

### Fractional atomic coordinates and isotropic or equivalent isotropic displacement parameters (Å^2^)

**Table:**

	*x*	*y*	*z*	*U*_iso_*/*U*_eq_	
S1	0.40383 (6)	0.31683 (4)	0.49763 (3)	0.04147 (14)	
S2	0.37003 (7)	0.28607 (4)	0.09053 (4)	0.04796 (16)	
N1	0.4334 (2)	0.52270 (16)	0.28512 (13)	0.0515 (4)	
H1A	0.4107	0.4478	0.3063	0.077*	
H1B	0.4850	0.5571	0.3302	0.077*	
H1C	0.5006	0.5166	0.2308	0.077*	
N1A	−0.0574 (3)	1.33594 (17)	0.21344 (16)	0.0635 (5)	
H1A1	0.0516	1.3376	0.1947	0.095*	
H1A2	−0.0772	1.3627	0.2736	0.095*	
H1A3	−0.1207	1.3845	0.1734	0.095*	
O1	0.4100 (2)	0.32750 (13)	0.60152 (11)	0.0566 (4)	
O2	0.5575 (2)	0.34399 (13)	0.44339 (12)	0.0595 (4)	
O3	0.2547 (2)	0.38716 (13)	0.46064 (14)	0.0666 (5)	
O4	0.5241 (2)	0.33961 (14)	0.05942 (14)	0.0723 (5)	
O5	0.3092 (2)	0.31235 (14)	0.18981 (12)	0.0669 (5)	
O6	0.2382 (2)	0.31961 (13)	0.02363 (12)	0.0615 (4)	
O1W	0.2991 (2)	0.49532 (17)	0.87440 (13)	0.0664 (4)	
H1WA	0.358 (3)	0.541 (2)	0.9040 (17)	0.079 (5)*	
H1WB	0.273 (4)	0.4416 (19)	0.9181 (15)	0.085 (5)*	
O2W	0.0816 (3)	0.5858 (2)	0.59992 (18)	0.0880 (6)	
H2WA	0.171 (2)	0.538 (2)	0.610 (2)	0.088 (5)*	
H2WB	0.014 (3)	0.542 (2)	0.578 (3)	0.111 (5)*	
C1	−0.0257 (2)	0.74496 (17)	0.22988 (14)	0.0439 (4)	
C2	0.0752 (3)	0.68758 (19)	0.15493 (14)	0.0485 (5)	
H2	0.0409	0.6979	0.0919	0.058*	
C3	0.2255 (3)	0.61548 (18)	0.17187 (14)	0.0457 (4)	
H3	0.2924	0.5783	0.1209	0.055*	
C4	0.2745 (2)	0.59965 (16)	0.26548 (14)	0.0404 (4)	
C5	0.1767 (3)	0.65344 (17)	0.34204 (14)	0.0450 (4)	
H5	0.2110	0.6411	0.4050	0.054*	
C6	0.0275 (3)	0.72579 (18)	0.32417 (14)	0.0466 (4)	
H6	−0.0388	0.7624	0.3756	0.056*	
C7	−0.1870 (3)	0.8271 (2)	0.21082 (17)	0.0532 (5)	
H7A	−0.2744	0.8101	0.2606	0.064*	
H7B	−0.2248	0.8081	0.1486	0.064*	
C8	0.3828 (2)	0.15788 (15)	0.48048 (12)	0.0361 (4)	
C9	0.2418 (2)	0.13005 (17)	0.43965 (14)	0.0434 (4)	
H9	0.1597	0.1931	0.4217	0.052*	
C10	0.2200 (3)	0.00641 (18)	0.42456 (15)	0.0459 (4)	
H10	0.1227	−0.0117	0.3974	0.055*	
C11	0.6604 (2)	0.08693 (16)	0.55070 (13)	0.0410 (4)	
H11	0.6764	0.1680	0.5620	0.049*	
C12	0.5117 (2)	0.06267 (15)	0.50798 (11)	0.0330 (4)	
C13	0.4159 (2)	0.12309 (16)	0.08655 (13)	0.0387 (4)	
C14	0.4905 (2)	0.06601 (15)	0.00027 (13)	0.0342 (4)	
C15	0.5455 (2)	0.13320 (16)	−0.08410 (14)	0.0424 (4)	
H15	0.5351	0.2190	−0.0839	0.051*	
C16	0.6129 (3)	0.07474 (18)	−0.16519 (15)	0.0521 (5)	
H16	0.6467	0.1208	−0.2199	0.062*	
C17	0.3680 (3)	0.05438 (18)	0.16698 (14)	0.0503 (5)	
H17	0.3225	0.0935	0.2230	0.060*	
C1A	−0.1634 (2)	0.96266 (19)	0.21010 (14)	0.0461 (4)	
C2A	−0.2043 (3)	1.0326 (2)	0.29072 (17)	0.0642 (6)	
H2A	−0.2545	0.9966	0.3461	0.077*	
C3A	−0.1734 (3)	1.1542 (2)	0.29229 (18)	0.0649 (6)	
H3A	−0.2018	1.1990	0.3480	0.078*	
C4A	−0.1006 (3)	1.20806 (19)	0.21109 (16)	0.0508 (5)	
C5A	−0.0614 (4)	1.1428 (2)	0.12952 (18)	0.0773 (8)	
H5A	−0.0133	1.1800	0.0740	0.093*	
C6A	−0.0929 (4)	1.0208 (2)	0.12897 (17)	0.0735 (7)	
H6A	−0.0660	0.9770	0.0726	0.088*	

### Atomic displacement parameters (Å^2^)

**Table:**

	*U*^11^	*U*^22^	*U*^33^	*U*^12^	*U*^13^	*U*^23^
S1	0.0483 (3)	0.0258 (2)	0.0497 (3)	−0.00183 (18)	−0.0032 (2)	−0.00024 (18)
S2	0.0611 (3)	0.0264 (2)	0.0561 (3)	−0.0029 (2)	−0.0017 (2)	−0.00822 (19)
N1	0.0616 (11)	0.0389 (9)	0.0524 (10)	0.0040 (8)	−0.0110 (8)	0.0015 (7)
N1A	0.0630 (12)	0.0455 (10)	0.0768 (13)	0.0069 (9)	0.0045 (10)	0.0060 (9)
O1	0.0774 (10)	0.0408 (8)	0.0526 (9)	−0.0095 (7)	−0.0006 (7)	−0.0113 (6)
O2	0.0672 (10)	0.0366 (7)	0.0731 (10)	−0.0118 (7)	0.0141 (8)	0.0023 (7)
O3	0.0683 (10)	0.0322 (7)	0.0996 (13)	0.0047 (7)	−0.0284 (9)	0.0023 (7)
O4	0.0768 (11)	0.0421 (8)	0.1011 (14)	−0.0221 (8)	0.0043 (10)	−0.0137 (8)
O5	0.0972 (13)	0.0412 (8)	0.0606 (10)	0.0018 (8)	0.0009 (9)	−0.0181 (7)
O6	0.0727 (10)	0.0348 (7)	0.0754 (11)	0.0048 (7)	−0.0159 (8)	0.0043 (7)
O1W	0.0779 (11)	0.0591 (10)	0.0653 (10)	−0.0166 (8)	−0.0188 (9)	0.0083 (8)
O2W	0.0815 (14)	0.0805 (14)	0.1024 (16)	−0.0080 (11)	0.0011 (12)	−0.0227 (12)
C1	0.0446 (10)	0.0385 (10)	0.0500 (11)	−0.0097 (8)	−0.0055 (8)	0.0018 (8)
C2	0.0612 (12)	0.0451 (11)	0.0396 (10)	−0.0037 (9)	−0.0107 (9)	0.0003 (8)
C3	0.0595 (12)	0.0370 (10)	0.0398 (10)	−0.0013 (8)	−0.0025 (9)	−0.0042 (8)
C4	0.0503 (11)	0.0273 (8)	0.0441 (10)	−0.0058 (7)	−0.0062 (8)	0.0004 (7)
C5	0.0588 (12)	0.0392 (10)	0.0382 (10)	−0.0089 (9)	−0.0079 (8)	0.0021 (8)
C6	0.0523 (11)	0.0438 (10)	0.0432 (10)	−0.0066 (9)	0.0027 (8)	−0.0036 (8)
C7	0.0434 (11)	0.0549 (12)	0.0615 (13)	−0.0049 (9)	−0.0071 (9)	0.0006 (10)
C8	0.0441 (10)	0.0274 (8)	0.0360 (9)	−0.0027 (7)	−0.0007 (7)	0.0002 (7)
C9	0.0471 (10)	0.0346 (9)	0.0478 (10)	0.0008 (8)	−0.0091 (8)	0.0008 (8)
C10	0.0455 (11)	0.0415 (10)	0.0527 (11)	−0.0062 (8)	−0.0142 (9)	−0.0031 (8)
C11	0.0474 (10)	0.0323 (9)	0.0446 (10)	−0.0075 (7)	−0.0060 (8)	−0.0039 (7)
C12	0.0404 (9)	0.0287 (8)	0.0293 (8)	−0.0037 (7)	0.0013 (7)	−0.0004 (6)
C13	0.0451 (10)	0.0274 (8)	0.0432 (10)	−0.0016 (7)	−0.0041 (8)	−0.0038 (7)
C14	0.0343 (9)	0.0268 (8)	0.0416 (9)	−0.0028 (6)	−0.0057 (7)	−0.0012 (7)
C15	0.0490 (11)	0.0273 (8)	0.0497 (11)	−0.0032 (7)	0.0008 (8)	0.0021 (7)
C16	0.0668 (13)	0.0393 (10)	0.0462 (11)	−0.0011 (9)	0.0083 (10)	0.0084 (8)
C17	0.0665 (13)	0.0398 (10)	0.0414 (10)	0.0027 (9)	0.0042 (9)	−0.0033 (8)
C1A	0.0366 (10)	0.0529 (11)	0.0465 (11)	0.0030 (8)	−0.0043 (8)	0.0057 (9)
C2A	0.0727 (15)	0.0602 (14)	0.0555 (13)	−0.0088 (12)	0.0233 (11)	0.0015 (10)
C3A	0.0736 (16)	0.0576 (14)	0.0583 (14)	0.0000 (12)	0.0213 (12)	−0.0067 (11)
C4A	0.0478 (11)	0.0427 (11)	0.0570 (12)	0.0091 (9)	0.0008 (9)	0.0079 (9)
C5A	0.120 (2)	0.0620 (15)	0.0465 (13)	−0.0132 (15)	0.0164 (14)	0.0118 (11)
C6A	0.111 (2)	0.0629 (15)	0.0436 (12)	−0.0113 (14)	0.0155 (13)	−0.0026 (11)

### Geometric parameters (Å, °)

**Table:**

S1—O2	1.4417 (16)	C7—H7A	0.9700
S1—O3	1.4495 (15)	C7—H7B	0.9700
S1—O1	1.4501 (15)	C8—C9	1.364 (3)
S1—C8	1.7900 (17)	C8—C12	1.432 (2)
S2—O4	1.4496 (17)	C9—C10	1.407 (3)
S2—O5	1.4507 (17)	C9—H9	0.9300
S2—O6	1.4531 (17)	C10—C11^i^	1.360 (3)
S2—C13	1.7759 (17)	C10—H10	0.9300
N1—C4	1.470 (2)	C11—C10^i^	1.360 (3)
N1—H1A	0.8900	C11—C12	1.418 (3)
N1—H1B	0.8900	C11—H11	0.9300
N1—H1C	0.8900	C12—C12^i^	1.431 (3)
N1A—C4A	1.478 (3)	C13—C17	1.369 (3)
N1A—H1A1	0.8900	C13—C14	1.428 (2)
N1A—H1A2	0.8900	C14—C15	1.419 (3)
N1A—H1A3	0.8900	C14—C14^ii^	1.431 (3)
O1W—H1WA	0.860 (10)	C15—C16	1.359 (3)
O1W—H1WB	0.854 (10)	C15—H15	0.9300
O2W—H2WA	0.847 (10)	C16—C17^ii^	1.401 (3)
O2W—H2WB	0.840 (10)	C16—H16	0.9300
C1—C2	1.389 (3)	C17—C16^ii^	1.401 (3)
C1—C6	1.399 (3)	C17—H17	0.9300
C1—C7	1.510 (3)	C1A—C2A	1.376 (3)
C2—C3	1.382 (3)	C1A—C6A	1.379 (3)
C2—H2	0.9300	C2A—C3A	1.379 (3)
C3—C4	1.375 (3)	C2A—H2A	0.9300
C3—H3	0.9300	C3A—C4A	1.366 (3)
C4—C5	1.378 (3)	C3A—H3A	0.9300
C5—C6	1.379 (3)	C4A—C5A	1.358 (3)
C5—H5	0.9300	C5A—C6A	1.384 (4)
C6—H6	0.9300	C5A—H5A	0.9300
C7—C1A	1.512 (3)	C6A—H6A	0.9300
			
O2—S1—O3	112.14 (10)	H7A—C7—H7B	107.9
O2—S1—O1	113.15 (10)	C9—C8—C12	120.94 (16)
O3—S1—O1	112.19 (10)	C9—C8—S1	118.27 (14)
O2—S1—C8	106.96 (8)	C12—C8—S1	120.78 (13)
O3—S1—C8	106.27 (9)	C8—C9—C10	120.28 (17)
O1—S1—C8	105.51 (8)	C8—C9—H9	119.9
O4—S2—O5	113.54 (11)	C10—C9—H9	119.9
O4—S2—O6	111.67 (11)	C11^i^—C10—C9	120.71 (18)
O5—S2—O6	111.49 (11)	C11^i^—C10—H10	119.6
O4—S2—C13	107.67 (9)	C9—C10—H10	119.6
O5—S2—C13	106.25 (9)	C10^i^—C11—C12	121.09 (17)
O6—S2—C13	105.67 (9)	C10^i^—C11—H11	119.5
C4—N1—H1A	109.5	C12—C11—H11	119.5
C4—N1—H1B	109.5	C11—C12—C12^i^	118.75 (19)
H1A—N1—H1B	109.5	C11—C12—C8	123.03 (15)
C4—N1—H1C	109.5	C12^i^—C12—C8	118.22 (19)
H1A—N1—H1C	109.5	C17—C13—C14	121.41 (16)
H1B—N1—H1C	109.5	C17—C13—S2	117.57 (14)
C4A—N1A—H1A1	109.5	C14—C13—S2	120.91 (13)
C4A—N1A—H1A2	109.5	C15—C14—C13	123.46 (15)
H1A1—N1A—H1A2	109.5	C15—C14—C14^ii^	118.9 (2)
C4A—N1A—H1A3	109.5	C13—C14—C14^ii^	117.66 (19)
H1A1—N1A—H1A3	109.5	C16—C15—C14	121.31 (17)
H1A2—N1A—H1A3	109.5	C16—C15—H15	119.3
H1WA—O1W—H1WB	103.5 (14)	C14—C15—H15	119.3
H2WA—O2W—H2WB	106.2 (15)	C15—C16—C17^ii^	120.51 (18)
C2—C1—C6	117.94 (19)	C15—C16—H16	119.7
C2—C1—C7	121.48 (18)	C17^ii^—C16—H16	119.7
C6—C1—C7	120.57 (19)	C13—C17—C16^ii^	120.20 (18)
C3—C2—C1	121.54 (18)	C13—C17—H17	119.9
C3—C2—H2	119.2	C16^ii^—C17—H17	119.9
C1—C2—H2	119.2	C2A—C1A—C6A	116.8 (2)
C4—C3—C2	118.83 (19)	C2A—C1A—C7	122.09 (19)
C4—C3—H3	120.6	C6A—C1A—C7	121.1 (2)
C2—C3—H3	120.6	C1A—C2A—C3A	122.3 (2)
C3—C4—C5	121.45 (18)	C1A—C2A—H2A	118.8
C3—C4—N1	119.68 (18)	C3A—C2A—H2A	118.8
C5—C4—N1	118.87 (17)	C4A—C3A—C2A	119.3 (2)
C4—C5—C6	119.20 (18)	C4A—C3A—H3A	120.4
C4—C5—H5	120.4	C2A—C3A—H3A	120.4
C6—C5—H5	120.4	C5A—C4A—C3A	120.2 (2)
C5—C6—C1	121.03 (19)	C5A—C4A—N1A	119.9 (2)
C5—C6—H6	119.5	C3A—C4A—N1A	119.9 (2)
C1—C6—H6	119.5	C4A—C5A—C6A	120.0 (2)
C1—C7—C1A	112.23 (16)	C4A—C5A—H5A	120.0
C1—C7—H7A	109.2	C6A—C5A—H5A	120.0
C1A—C7—H7A	109.2	C1A—C6A—C5A	121.5 (2)
C1—C7—H7B	109.2	C1A—C6A—H6A	119.3
C1A—C7—H7B	109.2	C5A—C6A—H6A	119.3

Symmetry codes: (i) −*x*+1, −*y*, −*z*+1; (ii) −*x*+1, −*y*, −*z*.

### Hydrogen-bond geometry (Å, °)

**Table:**

*D*—H···*A*	*D*—H	H···*A*	*D*···*A*	*D*—H···*A*
N1—H1B···O1^iii^	0.89	1.91	2.770 (2)	162
N1A—H1A1···O5^iv^	0.89	2.03	2.897 (3)	163
N1—H1C···O1W^iii^	0.89	2.07	2.948 (3)	167
N1A—H1A2···O2W^v^	0.89	1.86	2.739 (3)	171
N1A—H1A3···O1W^v^	0.89	1.95	2.807 (3)	161
O1W—H1WA···O4^iii^	0.860 (10)	1.801 (10)	2.645 (2)	166 (2)
O1W—H1WB···O6^vi^	0.854 (10)	1.957 (11)	2.807 (2)	174 (3)
N1—H1A···O2	0.89	2.46	3.007 (2)	120
N1—H1A···O5	0.89	2.47	2.996 (2)	118
N1—H1A···O3	0.89	2.49	3.125 (3)	129
O2W—H2WA···O2^iii^	0.847 (10)	2.68 (3)	3.072 (3)	110 (2)

Symmetry codes: (iii) −*x*+1, −*y*+1, −*z*+1; (iv) *x*, *y*+1, *z*; (v) −*x*, −*y*+2, −*z*+1; (vi) *x*, *y*, *z*+1.
